# Striving to promote male involvement in maternal health care in rural and urban settings in Malawi - a qualitative study

**DOI:** 10.1186/1742-4755-8-36

**Published:** 2011-12-02

**Authors:** Lucy I Kululanga, Johanne Sundby, Address Malata, Ellen Chirwa

**Affiliations:** 1 Department of International Health, Section for Health and Society, Faculty of Medicine, University of Oslo, P.O. Box 1130, Blindern, Oslo 0318, Norway; 2 Department of Community and Mental Health, Kamuzu College of Nursing, University of Malawi, P.O. Box 415 Blantyre, Malawi; 3 Department of Maternal and Child Health, Kamuzu College of Nursing, University of Malawi, Private Bag 1 Lilongwe, Malawi

**Keywords:** Malawi, male involvement, strategies, maternal health, care

## Abstract

**Background:**

Understanding the strategies that health care providers employ in order to invite men to participate in maternal health care is very vital especially in today's dynamic cultural environment. Effective utilization of such strategies is dependent on uncovering the salient issues that facilitate male participation in maternal health care. This paper examines and describes the strategies that were used by different health care facilities to invite husbands to participate in maternal health care in rural and urban settings of southern Malawi.

**Methods:**

The data was collected through in-depth interviews from sixteen of the twenty health care providers from five different health facilities in rural and urban settings of Malawi. The health facilities comprised two health centres, one district hospital, one mission hospital, one private hospital and one central hospital. A semi-structured interview guide was used to collect data from health care providers with the aim of understanding strategies they used to invite men to participate in maternal health care.

**Results:**

Four main strategies were used to invite men to participate in maternal health care. The strategies were; health care provider initiative, partner notification, couple initiative and community mobilization. The health care provider initiative and partner notification were at health facility level, while the couple initiative was at family level and community mobilization was at village (community) level. The community mobilization had three sub-themes namely; male peer initiative, use of incentives and community sensitization. The sustainability of each strategy to significantly influence behaviour change for male participation in maternal health care is discussed.

**Conclusion:**

Strategies to invite men to participate in maternal health care were at health facility, family and community levels. The couple strategy was most appropriate but was mostly used by educated and city residents. The male peer strategy was effective and sustainable at community level. There is need for creation of awareness in men so that they sustain their participation in maternal health care activities of their female partners even in the absence of incentives, coercion or invitation.

## Background

Traditionally, maternal health issues have predominantly been seen and treated as a purely feminine matter [1]. This was because women get pregnant and give birth. Although men's participation in maternal and child health (MCH) care services is low, they play a vital role in the safety of their female partners' pregnancy and childbirth. The exclusion of men from MCH services reinforced the erroneous notion that pregnancy and childbirth was uniquely feminine [2,3] and maternity units as exclusively meant for women [2]. It has however been discovered that some women's access and utilization of MCH services depend upon their partners [4].

A number of studies have highlighted the important role played by men in making decisions pertaining to maternal health issues and called for male involvement in MCH [4,5]. However, the men lack knowledge on maternal health issues that limits women's access to life saving treatment [6-8]. On the other hand, intervention studies have shown that maternal health education interventions targeting both men and women have proved to increase knowledge in both men and women [9-11]; increase health seeking behaviour among pregnant women [9,11]; raise awareness and use of family planning (FP) in the postpartum period, and also increase awareness of dual protection for STIs [12].

Effective strategies to reach men and engage them in sexual and reproductive health programmes are health facility, couple and community based. These strategies encourage men to attend antenatal and postnatal consultations where information on maternal health is provided to couples [9-12]. Men's participation in antenatal education programmes has positive effects including increase in men's reproductive health and child health knowledge, high utilization of antenatal care services by women.

In Malawi, pregnancy and childbirth has been women's domain and maternal health care services have focused on women, with very little male involvement. Literature has shown that attempts to involve men in maternal health care especially ANC, have managed to attract only few husbands [13-15]. Strategies that are used to invite husbands to participate in maternal health care by health care providers in different settings are seldom understood. Therefore, there is need to understand strategies that are used to invite men to participate in MCH. This information is important to formulate policies that remove barriers to male participation. This study investigated strategies that are used to invite men to participate in maternal health care in different health facilities in rural and urban settings in southern region of Malawi. While most male involvement studies targeted communities in rural and urban settings, this study has focused on the often-neglected strategies used by health care providers to invite men into the facilities. The aim of this study was to explore and describe strategies that are used to invite men into different health care facilities to participate in maternal health care of their female partners.

## Methods

### Design

The design of the study was descriptive non-experimental that utilized qualitative method. The data was collected using in-depth interviews that allowed in-depth exploration of the under-researched area of the perspectives of health care providers on strategies that are used to invite men to participate in maternal health care [16]. The study was part of a major project that is focusing on male involvement in maternal health care in rural and urban settings in Malawi.

### Settings

Data was collected from five different health facilities in the southern region of Malawi. The sampled health facilities comprised two health centres, one district hospital and one central hospital owned by government. Two more health facilities, one belonging to the Christian Healthcare Association of Malawi (CHAM) and one privately owned were also included in the sample. The health care facilities were purposively chosen in order to capture views from health care providers from different health care settings. The government owned facilities were Zomba Central Hospital (ZCH), Mwanza District Hospital (MDH) and its affiliated health centres (Kunenekude and Tulonkhondo). The CHAM facility was Mlambe Mission Hospital (MMH) and the privately owned facility was Blantyre Adventist Hospital (BAH).

**Zomba Central Hospital **is situated 65 kilometres east of the city of Blantyre. It is a teaching hospital and offers tertiary level services. In addition, it is a referral hospital for 4 surrounding districts and 28 health centres. Zomba city has a university campus, but no district hospital and all referrals from health centres come straight to the central hospital. It is an 800 bedded hospital and provides specialized obstetric and gynecological services. The obstetric part is managed by obstetricians, and general practitioners (medical doctors and clinical officers), State Registered Nurse Midwives (SRNMs) and Nurse Midwife Technicians (NMTs). The hospital also offers routine MCH services to the people around its catchment area. Male involvement in maternal health care is encouraged especially in ANC, postnatal, family planning and prevention of mother to child transmission (PMTCT) of HIV services.

**Mwanza District Hospital **is located 100 kilometres north of Blantyre City. The hospital serves a population of 93, 000, with an annual growth of 4.1%. The district hospital serves as a referral hospital to the three health centres and dispensaries within its catchment area. The district hospital also receives referrals from health facilities in Chikwawa district with which it shares boundary. Furthermore, it receives a considerable number of patients from border villages of Mozambique and referrals from health centres in Mozambique. It also collaborates with non-governmental organizations (NGOs) within the district. The district hospital provides comprehensive emergency obstetric care (CEmOC). General practitioners, SRNMs and NMTs provide maternal health care services.

Data for this paper were also collected from two of the district's health centres namely Kunenekude and Tulonkhondo. Kunenekude health centre is 19 km north of the district hospital and it serves an estimated population of 15,800, while Tulonkhondo health centre which is 17 km south of the district hospital serves an estimated population of 14,500. The health centres provide basic emergency obstetric care (BEmOC). Two NMTs at each health centre provided maternal health care services.

Male involvement in reproductive health was initiated in Mwanza district by Ministry of Health with the support from UNICEF in 2004. UNICEF pioneered a project called 'Male Champion Initiative' and the aim of the project was to involve more men in their partner's reproductive health. Men were encouraged to accompany their wives to antenatal clinics where services such as PMTCT are offered.

**Mlambe Mission Hospital **is situated 30 kilometres north of Blantyre city. It is a 254-bed facility run by the Roman Catholic Church. The hospital is one of the Christian Hospital Association of Malawi (CHAM) facilities. CHAM is an ecumenical, not for profit non-governmental umbrella organization of Christian owned health facilities. CHAM offers about 37% of health care services in Malawi [17]. Ninety percent of CHAM health facilities are located in the rural settings of the country where, in most cases, there are no government facilities. Thus, Ministry of Health signed a contract with CHAM to provide free maternal healthcare through Service Level Agreements (SLAs). Mlambe Mission Hospital signed a SLA contract with Blantyre District Health Office to enable it provide maternal health care services to the people around its catchment area. In addition, the hospital receives obstetric referrals from six government health centres. The hospital offers ANC, labour and delivery and postnatal care. It does not offer family planning services due to this church's guiding principles. Family planning clients are referred to government and other non-governmental facilities that offer the services. An obstetrician, general practitioners, clinical officers, SRNMs and NMTs, offer the MCH services. The hospital policy also encourages male involvement in maternal health care.

**Blantyre Adventist Hospital **is located at the centre of Blantyre city. The American missionary doctors of the Seventh-Day Adventist Church established the hospital in 1957. It is a forty bedded private-for-profit hospital. It offers specialized obstetric care and operated by an obstetrician, SRNMs and NMTs. Since its inception, the hospital encourages men to participate in maternal care of their wives/partners, including attendance at delivery.

### Participants and recruitment

Twenty health care providers were approached to participate in the study. However, the final sample comprised sixteen health care providers because four refused to participate. The reasons for refusal given by health care providers included being busy with their work and did not want to use their free time for the interviews; and being tired of participating in students' research. The participants were purposively selected among the health care providers. Health care providers who expressed willingness to participate in the study were Malawians working in the MCH department for not less than 6 months. Thus, the selection of participants with individual experience in MCH and male involvement was purposeful in order to ensure credibility of results. Written informed consent was obtained and the health care providers were guaranteed of their confidentiality and freedom to withdraw from the study at anytime. They were also informed that they were free not to answer any question that they felt not comfortable to answer. The participants' age ranged from 21 to above 50 years and four of the participants were male. Professional qualifications included SRNM, NMT and clinical officers. Furthermore, their experience in MCH ranged from 1 to 25 years. Of the 16 participants, 3 were from Blantyre Adventist Hospital, 5 from Mwanza District Hospital, 4 from Mlambe Mission Hospital and the other 4 from Zomba Central Hospital. Recruitment stopped after achieving data saturation [18].

### Ethical consideration

Malawi College of Medicine Research and Ethical Committee and the Regional Committee for Medical and Health Research Ethics in Norway approved the study. In addition, permission to collect data was obtained from the directors of the sampled facilities and written informed consent was obtained from individual participants.

### Data collection

A semi-structured questionnaire was administered to 16 individuals that consented to participate in the study. The structured part collected participants' demographic data and the open-ended part captured qualitative data. The interviews were held in Chichewa and lasted between 40 to 60 minutes. The health facility management provided a private office for the interviews. All interviews were audio-recorded except for two participants who refused to have their responses audio-taped. For these two participants, hand written notes were used to record responses. Field records were taken for all participants' responses and the hand written notes were expanded into transcripts.

### Data Analysis

Data analysis was undertaken simultaneously with data collection in order to identify and correct errors during next interviews. The taped information was transcribed verbatim and translated from vernacular language into English. Thematic content analysis guided data analysis [19]. The transcripts were read repeatedly and words with similar meanings were grouped into categories using Nvivo 9 software. Similar categories were grouped into themes and sub-themes that are presented as results. The results contain direct quotes from participants and narrations are reported as spoken by participants without editing the grammar to avoid losing meaning. Expressions in vernacular language are presented in parentheses and fictitious names are used in quotes to maintain confidentiality of the participants.

## Results

### Participants' demographic characteristics

The participants' age ranged from 21 to above 50 years and four of the participants were male. Professional qualifications included SRNM (2), NMT (13) and clinical officer (1). Furthermore, their experience in MCH ranged from 1 to 25 years. Of the 16 participants, 3 were from Blantyre Adventist Hospital, 5 from Mwanza District Hospital, 1 each from the two health centres, 4 from Mlambe Mission Hospital and the other 4 from Zomba Central Hospital.

The participants' responses generated four main strategies that were used to invite men to participate in maternal health care services. The strategies were health provider initiative and partner notification that were at health facility level, while the couple initiative was at family level and community mobilization at village (community) level. The community mobilization had three sub-themes namely; male peer initiative, use of incentives and community sensitization.

### Health care provider initiative

In this strategy, the health care provider asked the pregnant woman to bring her husband next time she comes for ANC services. The woman decided whether to invite the husband or not. On the other hand, when the woman invited her husband to participate in her care at the ANC, the husband decided whether to participate or not. When the husband decided to participate, he accompanied the wife during the subsequent ANC visits. This strategy was also used to solicit husband participation in the PMTCT programme. The health care providers further explained that couple counseling and HIV testing is encouraged in order to promote women's adherence to PMTCT interventions. Mandatory HIV test is part of the routine antenatal care in Malawi. Hence, the health care providers encouraged couple counseling because it was noted that many women were not disclosing their HIV status to their spouses out of fear of their partners' reaction. The health providers were of the view that inviting men to the ANC clinic where HIV testing is part of the routine has its own challenges. The coming of the husband to the clinic depended upon the wife's freedom of choice and her perception of the benefits of having a husband participate in the facility based maternal care. The social association of HIV positive status and infidelity hindered many women from inviting their husbands to the ANC.

"When a woman comes to the clinic for the first ANC, we ask her to come with a husband during the next visit. The husband is needed so that we can counsel the couple on how to prepare for the baby and on HIV testing and prevention of mother to child transmission of HIV (PMTCT). Some men do come during the next visit." (Jane)

Another provider initiative at facility level involved giving first and fast service to women that attended ANC with their couples. The heath care provider interviews showed that men as main family breadwinners could not manage to postpone or stop working so that they participate in maternal health care of their partners. Husbands/partners who were employed needed to ask for some hours off duty to accompany a wife for antenatal care. Those husbands, who earned a living by small-scale business like selling vegetables, felt that they wasted time by being at ANC instead of attending to personal business that brought money to the family. To attract men to participate in maternal health care, the providers introduced the first and fast service for couples so that the husbands/partners should not waste time on the queue at the facility. First and fast service strategy was used in all the health facilities where this study was conducted except BAH.

"Another thing is that most men are busy, so when they come I think it can be a good thing if we attend to those who come with their husbands first, so that next time they can come knowing that they will be helped fast and they won't be late for work or any of the activities that help them to earn a living." (Mable)

Women were encouraged to come with their husbands to the clinic and were assured of a fast and first service. This initiative also worked well for the women because they did not spend much time at the clinic and they went back home in good time to do other activities. The health providers perceived the use of this type of initiative as unsustainable.

"It is attractive now because few men are attending ANC. It may not be applicable when almost all women would be accompanied by husbands."(John)

### Partner notification

Partner notification is another provider-initiated strategy, which is used to solicit male participation when a woman has a sexually transmitted infection (STI). The main purpose for this strategy is to have the husband/partner treated for STI in order to control the infection. Some of the STIs, such as syphilis do have adverse effects on the foetus as well. In partner notification, the provider explained to the woman the importance of having a husband/partner treated for the infection. Then, the woman was given a notification card to give to the husband. The notification card had the woman's identification number and the husband/partner had to present this card to the health care provider at the clinic. The husband could come alone to the ANC or with the wife. However, the health care providers preferred that the couple be together for further counseling. The view that men were being invited in maternal health care portrayed them as second level clients who could only attend the clinic when there was pathology. On the other hand, pregnancy justified women's use of the services.

"Sometimes we do call for the husband/partner when the woman has problems that require counseling to both of them, for example when a woman has sexually transmitted infection. The treatment requires that the man be treated as well and they should abstain from sex until they have finished the treatment. In such cases we call for the man if he was not coming for antenatal care together with the wife." (Jane)

The providers noted that almost all men who were invited to the clinic through partner notification came for the treatment. However, they did not come back to the clinic when they have finished the treatment. The majority of the health care providers acknowledged that husbands were being invited to participate in specific maternal health interventions such as HIV testing and STI treatment. They viewed that using this strategy limited male participation to specific maternal health services as such husbands did not see the need to accompany the wives to the clinic when there was no agenda for them. Partner notification strategy was used in all the health facilities where this study was conducted.

### Couple initiative

In this strategy, the couple agreed to jointly participate in maternal health care. Most husbands participated in maternal health care when a wife conceived after a long waiting time. In this case, the pregnancy was precious and the husband was anxious about the well-being of the foetus and the mother. As such, the husband was very interested to know the progress of the pregnancy and the health of the wife. The health care providers described such husbands as very inquisitive, wanting to know what was happening to the foetus and the wife. They asked about the results of every test done on the wife and about the resultant treatment. The participants also mentioned that young educated couples used this strategy regardless of whether it was a low or high-risk pregnancy. In addition, the health care participants described the couples who used this strategy as educated, exposed to male involvement information through the mass media, internet and had travelled abroad. The participants described that the couples who used couple initiative strategy attended all ANC visits together and the husband was present during labour and delivery. The husband also accompanied the wife during postnatal care consultations. This strategy was commonly used at BAH and Mlambe Mission Hospital (private wing) due to the fact that the labour wards in these facilities offered some privacy and that the facilities were accessed by educated and city residents.

"Most of our clients are educated and well to do. They are used to men being allowed in ANC consultations, labour and delivery wards and postnatal consultations. Previously, non-Malawian men were the ones that used to accompany their wives for maternal health care services. But nowadays, we are seeing a lot of Malawian men coming to the clinic with their wives, and some even assisting the wife in the labour ward." (Martha)

"Nowadays most men are participating in maternal health care. Last month [October 2010] there was a woman who was in labour and admitted in the private labour ward. The husband was present throughout labour and delivery supporting the wife. There was a time when the woman was in early labour and was asked to be walking around. I saw the couple holding hands walking around the hospital. For me this is male involvement."(John)

However, at Zomba Central Hospital there were also a few couples who used this strategy. The husbands participated in all ANC and postnatal consultations but not in labour and delivery care due to privacy issues. The hospital had one big labour and delivery room with 10 delivery beds that were demarcated by curtains. Most often than not, women labour naked, as the hospital did not have hospital gowns and women had to spare their home clothes to be put on after delivery. The lack of privacy in the labour room hindered birthing women to have a spouse or family member present in labour ward for emotional support.

### Community mobilization strategy

#### Male peer initiative

Men who had participated in maternal health care inform their peers about their experiences. The other men become motivated and discussed with their wives. When they agreed, the couple visited the ANC together. The husbands would decide whether to be present during labour and delivery.

"....another man told me that he learnt about male involvement from a friend. He came to the clinic with his wife just to see what was there for men. At the end of the clinic, he said that he had learnt a lot about pregnancy issues. He also appreciated the way he was welcomed at the clinic." (Jane)

The health care providers were of the view that male peer approach should be emphasized, as men would want to identify with fellow men. So the men that are influential among their peers should be targeted with male involvement information in order for them to be role models for their peers.

#### Community sensitization

The health care providers at Mwanza District Hospital and its affiliated health centres mainly used community sensitization strategy. The health care providers felt the need to involve men in maternal health care. They approached the traditional authorities and chiefs, who are highly respected community leaders in Malawi, and were informed about the maternal health problems in the district. The health care providers in collaboration with the community leaders developed plans on how to involve men. Different methods were used such as community outreach, public meetings, use of incentives, and launching of male involvement. The collaboration with the community leaders also enabled the health care providers to access the communities and households with male involvement information.

"We involved the village chiefs to disseminate the information about male involvement in safe motherhood. Firstly, we talked with the chiefs about maternal health problems that women in the area are facing such as poor health and maternal deaths. Then, we would discuss with the chiefs on strategies that can be used to prevent maternal deaths and improve maternal and child health. Then we would introduce male championship as one of the strategies and encourage the chiefs to motivate the men to accompany their wives for antenatal care, especially the first visit."(Ruth)

Health surveillance assistants (HSA) who are the community health care workers in Malawi mainly conducted community outreach. The HSAs spread the male involvement messages in the villages. They collaborate with the community leaders on how to approach the men. The villagers do identify with the HSAs because they live in the same villages and the communities respect them. Due to the nature of the work of the HSAs, they do interact with the villagers and do know the socio-cultural norms of the communities in which they work. This enables them to disseminate the male involvement information in a socio-culturally acceptable manner. The community members felt free to ask them for clarification on any information to do with maternal health and male involvement.

"Probably the campaign on safe motherhood has made a difference. Here at Mwanza District Hospital there is a safe motherhood team. This team goes into the villages encouraging men and women on the importance of antenatal care, hospital delivery, and family planning. They also emphasize the importance of male involvement in these issues. Since the initiation of male championship in this district, we have seen a change in health care seeking behaviour of the people. Most women are delivering here at the hospital. In addition, we have also seen a drop in maternal deaths." (Pamela)

Public meetings were another avenue that community leaders use to disseminate information about male involvement in maternal health care. The information was disseminated during political meetings, funerals and social gatherings. The health care providers were of the view that the community leaders were motivated to do this due to the incentives they were getting and the perceived benefits for women, families and the community in general. However, the health care providers explained that during public meetings, the community members could not ask questions to the traditional leaders. In this community, it is regarded as lack of respect to ask a leader or an elderly person questions in public. However, the health care providers felt that the information that was received during public meetings stimulated much debate as well as communication between couples and households. This was evident when some men sought for more information from the health care providers about male involvement because of such meetings.

"*The group village headman here is very much into it. He takes opportunity of every public meeting, be it political, religious or even at a funeral, to talk about male involvement. He tells the people that it is one of the important strategies to reduce maternal and child deaths." (Peter)*

#### Use of incentives

At community level, Mwanza District Hospital used competition among villages to encourage male involvement in maternal health care. UNICEF organized the competition in 2008. A traditional authority and village chief that had high proportion of couples attending antenatal clinic received a prize. The use of the incentives motivated village chiefs to become vigilant in promoting male involvement. The village chiefs advised health care providers not to attend to any woman who came to the antenatal clinic without a husband in order for them to get a prize.

"In this competition, the organizers were looking at the number of couple against the total number of mothers who came for antenatal visit..... The village headman for that particular village (with a high proportion of couples would) receive a bicycle. In addition, the Traditional Authority with high proportion of couples would receive a bicycle and a trophy. These incentives motivated the chiefs to disseminate the male championship messages to the villagers. Some chiefs went to the extent of advising the health care providers not to attend to any woman who came for antenatal care without a husband. If the husband is away, the woman was supposed to get a letter from the chief." (Ruth)

However, the health care providers recognized that sending women back who did not come to the antenatal clinic with a husband was not professionally acceptable. They did it out of respect for the traditional authorities, importance of the initiative and saving a collaborative relationship with the communities.

The health care providers explained further that when the competition came to an end there was a decline in the number of couples attending ANC services. The health care workers viewed that the competition was a motivating factor for the men to participate in maternal health care. However, the strategy failed to induce behavioural change towards male participation in facility based maternal health care.

"In fact this practice was only effective in 2008 because of the competition. Now there are no longer material rewards but still the people are used to the principles of the initiative. However, few men do accompany their wives for antenatal care." (Ruth)

Women were encouraged to come with their husbands to the clinic and were assured of a fast and first service. This incentive also worked well for the women because they do not spend much time at the clinic and they went back home in good time to do other activities. The health care providers perceived the use of this type of incentive unsustainable.

"It is attractive now that few men do attend the ANC. It may not be the case when almost all women would be accompanied by husbands." (John)

The only hospital that did not use incentives to motivate male participation was BAH. The clients booked for antenatal consultation and were given a specific time when to see the doctor. In the other study sites, they use the first come first served kind of model. Hence, women queued for the services, and women that were accompanied by their spouses were attended to immediately without queuing.

#### Sensitization campaigns

Sensitization campaigns helped in Mwanza district to encourage men to participate in maternal health care. The health providers used a number of strategies to sensitize the communities about male involvement such as launching the programme and community outreach. The male involvement programme was launched publically in the district. During the launch, people were entertained with drama and traditional dances that conveyed male involvement and maternal health messages. The District Health Officer and one of the Traditional Authorities for the district made speeches to emphasize the importance of the programme. The presence of the traditional authorities at the function signaled the leaders' approval and commanded an obligation from the part of the community members.

Mwanza District Hospital had a safe motherhood team that went out into the villages to disseminate information and provided reproductive health services. One of the messages that were disseminated was male involvement. The health team focused on providing information about male involvement in relation to HIV testing and PMTCT interventions. Couples were encouraged to go to the health facilities for couple counseling and HIV testing. Men were told that they were only needed at the ANC during the first visit when the HIV test was done. The health care workers said that they had noted a difference in health care seeking behaviour of the men in the areas they had visited. In addition, they also said that they had noted an increase in hospital deliveries and reduction in maternal deaths.

"Probably the campaign on safe motherhood has made a difference. Here at Mwanza District Hospital there is a safe motherhood team. This team goes into the villages encouraging men and women on the importance of antenatal care, hospital delivery, and family planning. They also emphasize the importance of male involvement in these issues. Since the initiation of male championship in this district, we have seen a change in health care seeking behaviour of the people. Most women are delivering here at the hospital. In addition, we have also seen a drop in maternal deaths." (Pamela)

## Discussion

### Health care provider initiative

Health care provider initiative strategy depended on the health care provider to initiate the invitation of husbands to participate in maternal health care. This strategy was used in all the five study sites. Legitimate power is provided to the women with this strategy. The women exercise freedom of choice of whether to invite the husband/partner or not. Kabeer describes power in terms of the ability to make choices [20]. By giving women the freedom to invite their husbands/partners, the health care provider seemingly, allow for a degree of autonomy on behalf of the women. However, in order for the women to be truly empowered, there is need for an ability to formulate one's own preferences and to look for alternatives. Thus, health care providers have the responsibility of explaining the reasons for inviting the husbands to the clinic and the impact that it will have on the woman's health care. This information is important for the woman to make an informed decision. Unless the pregnant women are empowered with a real informed opportunity to make choices, they may not invite their spouses for a variety of reasons deeply embedded in their own culture. Other studies done in Malawi describe lack of knowledge on the importance of ANC and that some of the women attend ANC to get a card to avoid being scolded by health care providers during labour [21]. For such women it may be difficult to understand the importance for male involvement and let alone inviting them to participate in the care. Furthermore, for men who may view maternal health care services as feminine may decide not to accompany the wife in order to maintain their masculine power despite being invited. The invitation is still seen as a recommendation from the side of public services, not as a deeply felt choice that is eventually effectuated.

The health care providers indicated that the routine antenatal care of the women is not affected if the husband/partner is not present. They further expressed that male participation is very low compared to the number of women that attend antenatal care in the respective health facilities. However, the health care providers attributed low male participation to men's commitment to income generating activities for their families and lack of knowledge of male involvement programme. Theuring also found similar results in the study done in Mbeya Region, Tanzania [22]. Men's lack of knowledge about male involvement programmes, and a lack of priority for such activities when competing with other challenges, could be attributed to the strategies used to disseminate the information.

Health care provider initiative strategy is also dependent on the attitudes of the health care provider towards male involvement. The health care providers that have positive attitude towards the programme would encourage the women to invite their partners. They would take time to explain to the women the benefits of having a husband/partner involved in their care. Furthermore, when the husband accompanied the woman the provider would do her best to attend to the couple and make the visit worthwhile to both the woman and the man. However, poor health care providers' attitudes have been attributed to low male participation in Malawi [23].

### Partner notification

The fact that partner notification was used when a woman was diagnosed with an STI could mean that this strategy was not "male involvement" per se, nevertheless, draws men into the services. However, inviting men to participate in maternal health care services through partner notification portrays them as second level clients who could only benefit directly from its services when there is pathology. On the other hand, pregnancy justifies women's use of the services. Aarnio and friends [13] report similar findings.

Partner notification has been used in STIs control but its effectiveness in male involvement in the whole menu of services has not been explored in Malawi. However, evidence shows that there is low effectiveness of partner notification with STIs [24,25]; and that partner notification alone without community mobilization does not suffice to attract men [26,27]. Nevertheless, anecdotal report from Mulago hospital staff in Uganda indicate that use of invitation letters has increased male participation during antenatal and postnatal care of women from the national average of 7% to 15% [28]. At Mulago Hospital in Uganda, all mothers visiting ANC, regardless of HIV status, were given invitation letters for their spouses [28].

The dependency on women to invite men for male involvement in maternal health care indicates lack of readiness by the health care sector to invest in community based approaches that have proved successful elsewhere [29]. In this study, the health care providers in the MCH department were often overburdened with their work. Some of the health care providers expressed that it was not possible for them to engage in active male involvement campaigns. The Zomba Central Hospital health care providers alluded to that fact that the hospital is a referral facility. Community based activities were supposed to be done by Zomba District Health Office. The only strategy they could use was to encourage male participation through the women who came for services at the hospital. However, it was noted that there was lack of coordination between the two health departments. The Zomba Central Hospital health care providers were not aware of male involvement activities that the District Health Office engaged in. The health care providers at Blantyre Adventist Hospital indicated that they encouraged male participation but did not conduct community-based campaigns. It was the responsibility of Blantyre District Health Office. However, the health care providers indicated that they could conduct outreach services when funds were available. Similar sentiments were expressed by Mlambe Mission Hospital Staff. On the contrary, Mwanza District Hospital had health care providers conducted community based male involvement campaigns. However, the results of such campaigns have brought much impact at health centre level than at district hospital level. The positive impact at health centre level could be attributed to the fact that most of the men that live within the catchment area of the health centres were peasant farmers who could easily be mobilized.

### Couple initiative

Couple initiative strategy was found to be a strategy commonly used by couples who visited BAH and Mlambe Mission Hospital private sections. Most of the clients who used these facilities were from the affluent sector of the society. Feyisetan found similar results in Nigeria where by education played a significant role in spouse communication [30]. They found that spouse communication about contraceptive use was greatly enhanced when both spouses had similar levels of education (or close to one another). The significance of education was much more pronounced when none of the partners had below secondary education and at least one of them had a post- secondary education [30]. It should be noted that such type of couples have access to mass media, internet and exposure to the outside world, factors that influence behavior change. This strategy was most efficient and sustainable but unfortunately only confined to the few educated and urban dwellers.

Spouse communication was associated with the empowerment of women within the marital union. A number of factors indicate the empowerment of women and the increase of their status within the household and the marital union. Feyisetan posits that at higher levels of education and with little difference in educational attainment, partners appear to feel more comfortable discussing issues which are traditionally thought to be under the control of men [30]. Thus, education and exposure to the western culture influence couple's attitudes and behaviour towards increased gender equity, expressed as shared responsibility for the pregnancy and the baby.

### Male peer initiative

The results of this study have shown that informal peer information giving is one of the strategies that are used to invite men to participate in maternal health care. Men who have participated in maternal health care inform peers about their experiences and encourage them to participate. Zulu asserts that men in Malawi, whether in a patrilineal or matrilineal social system, are brought up to believe that they are inherently superior to females and therefore tend to downplay the importance of new ideas originating from females, especially issues of reproduction which affect a man's social status[31]. Thus, men may be more receptive to maternal health information originating from fellow men than women. Similar finding are reported by Aarnio and friends in a study done in Malawi on male involvement in antenatal HIV counseling and testing; Elizabeth Glaser Pediatric AIDS Foundation in a study done in a rural community in Tanzania that explored the role of male participation in PMTCT programs; and Onyango and friends in Kenya [13,28,32]. In addition, Avogo and friends found that men and women's discussions in gendered networks are significantly associated with subsequent spouse communication about family planning [33]. They further posit that social influence is directly reflected when informal social networks exchange information on childbearing [33].

It is worthy noting that in some Malawi societies, boys and girls are socialized into manhood and womanhood through separate initiation ceremonies that are conducted in separate huts and are encouraged to keep secret from the opposite sex and the uninitiated. Furthermore, men have their own place where they spend their leisure time after the day's work where they do discuss men's issues. Similarly, women have their own designated place where they spend their leisure time socializing. Either places, men's or women's are no go areas for the opposite sex. If either sex is found in the other sex place are ridiculed called names such as *Chili pa akazi *(feminine) or *Chili pa amuna *(tomboy) [34]. It is in these gendered spaces that informal education of boys and girls, men and women takes place. Such gendered social norms make any other form of information sharing a "deviant form", that is, an attempt to break strong social gender barriers - and may at this stage be counter-productive as they ask for something that the community is not ready for yet.

### Community mobilization

Community mobilization strategy was mainly used by the health care providers at Mwanza District Hospital and its affiliated health centres. The health care providers in collaboration with traditional and community leaders mobilize the community in general and men in particular, for male involvement. The community leaders are gatekeepers of their communities. The cooperation of community leaders is very crucial in the advancement of any public health initiative in Malawi. The effectiveness of collaboration with traditional authorities has been evident in reducing maternal mortality in some district of Malawi [35]. In rural areas of Mwanza District, community outreach is mainly conducted by HSAs who are the primary community workers in Malawi and they do reside in the same communities. The health care providers in Mwanza District pointed out that the community approach is feasible in rural areas where the community members can easily be mobilized by the traditional leaders. Working with traditional leaders can be very fundamental because of their power position within the community. In communities where the traditional leader is a man, he can be the agent of change among men, as he may act as a role model.

### Sensitization campaign

Sensitization campaigns were another strategy that targeted communities at Mwanza District Hospital. The district used educational messages combined with entertainment as they were launching the male involvement programme. The power of entertainment-education to effectively promote change in health related beliefs and behaviour is well-documented [36]. The sensitization campaigns raised awareness, stimulated discussion among peers and couples about male involvement leading to the desired action. The health care provider expressed that some of the community members sought for more information about male involvement following the sensitization campaigns. The dilemma is that campaigns are often short lived, and may not initiate enough change to enhance a long-term behaviour change.

### Use of incentives

Fast and first service is the incentive that is being used in all antenatal and postnatal clinics in the study sites but for BAH. Similar findings were also cited by Elizabeth Glaser Pediatric AIDS Foundation [28]. It should be noted that at BAH, women had to book for an appointment with an obstetrician and were given a specific time for the appointment. Thus, there was no need for preferential treatment whether one has come with a spouse or not. On the other hand, the other health facilities had a tradition of having all the women congregating at 07:30 hours (the official time to start work in Malawi). The clinic commenced with a group health educational talk, which could last 30 to 60 minutes depending on the topic under discussion and the person giving the talk. It was during this group health educational session that male involvement promotional talk was also given. Then, the clients went for HIV counseling and testing if it was the first visit. The women queued for blood pressure and weight check, and physical examination. Thereafter, they had to queue for medication (malarial prophylaxis and iron tablets). Depending on the number of the clients that were available on that particular day and the processes that a woman had to undergo, she could spend 2 to 4 hours at the clinic. Consequently, first and fast services were an incentive when women came with their spouses.

The waiting time was an issue when it came to dealing with men. The health care providers expressed that they offered this kind of incentive in order to release the man to go back to his work. It might imply that for men time is money and women have all the time to spend at the clinic. This practice reflects the traditional gender role norm that men are breadwinners and that they should not spend a lot of time at the clinic. This strategy may not be sustained in the near future when more men accompany their wives and queuing becomes inevitable. However, the strategy has managed to stimulate men to accompany their wives to facilities.

### Competitions

Mwanza District Hospital used competitions among communities in different traditional authorities to advance the male involvement programme. The health care providers alluded to the fact that there were more couples attending the ANC during the competition period and the numbers dropped when the competition ended. There are several possible reasons for the decline in couple ANC attendance after the competition. Firstly, couples may have been pressurized to attend ANC because of the competition. The competition was the driving force. When the force was removed, the men did not have the will to continue. Secondly, the main emphasis of the competition was male involvement in HIV testing and PMTCT. When the men knew their HIV status, there was no need for them to go to the clinic even for subsequent pregnancies. This was equally true with men in a polygamous marriage. The men felt no need to accompany the other wives when they knew already their HIV status. Thirdly, after the competition, the health care providers did not feel obliged to force women to come to the clinic with husbands. Having men at the clinic implied additional workload for the health care providers. Therefore, competitions like other incentives were not sustainable in the long run to promote male involvement in maternal health care services.

### Limitations of the study

The main limitation of this study is reporting bias arising from participants wanting to provide socially desirable responses rather than true reflection of the real life situation. The participants were aware that the interviewer was a nurse-midwife and that may have influenced the information given.

Furthermore, in this study four health care providers declined to participate as provided for in the consent form. It may be postulated that had they participated further items on strategies to invite men to participate in maternal health care could have been identified. However, it might have also been not so from the position that providers were using the same methods and any one group could have provided the same information.

## Conclusions

This study shows that it is possible to involve men in maternal health care. Participants in this study used facility, family and community based strategies to involve men in maternal health care. At facility level, the strategies used were not sustainable although they increased male participation in the short run. A major constraint was the fact that male participation depended upon the wife's willingness to deliver the invitation from the health care providers and the husband's willingness to participate.

Couple based initiative was more effective and sustainable because it originated from the couple itself that felt a need to jointly get involved in maternal health care services. This strategy however, was mostly adopted by the educated and city residents.

Community mobilization strategies were more effective to rural settings in terms of coverage. The male peer strategy was both effective and sustainable, should hence be encouraged, and promoted. Other community-based strategies worked well where there were incentives. The incentives were however donor driven, thus they only worked well during the project lifetime. It is therefore recommended that while all the strategies are encouraged, more emphasis should be made on couple and male peer strategies to sustain male participation in maternal health care. There is also a need for long-term strategies targeting a whole generation in order to bring the desired behaviour change in male involvement towards maternal health care.

## List of abbreviations used

ANC: Antenatal care; BAH: Blantyre Adventist Hospital; BEmOC: Basic emergency obstetric care; CEmOC: Comprehensive emergency obstetric care; CHAM: Christian Hospital Association of Malawi; FP: Family planning; HIV: Human immunodeficiency virus; HSAs: Health surveillance assistants; MCH: Maternal and child health; MDH: Mwanza District Hospital; MDHS: Malawi Demographic and Health Survey; MMH: Mlambe Mission Hospital; NGOs: Non Governmental Organizations; NMTs: Nurse-Midwife Technicians; PMTCT: Prevention of mother-to-child transmission; SLAs: Service Level Agreements; SRNMs: State Registered Nurse-Midwives; STI: Sexually Transmitted Infections; UNICEF: United Nations Children's Fund; ZCH: Zomba Central Hospital.

## Competing interests

The authors declare that they have no competing interests.

## Authors' contributions

LIK conceptualized the study, collected the data, led the analysis, and wrote the text of the paper. JS, AM and EC advised on the conceptualization of the study, analysis of the data, and presentation of the results, review and edited the text. All authors read and approved the final manuscript.
